# Antiresorptive Versus Anabolic Therapy in Managing Osteoporosis in People with Type 1 and Type 2 Diabetes

**DOI:** 10.1002/jbm4.10838

**Published:** 2023-10-29

**Authors:** Tatiane Vilaca, Richard Eastell

**Affiliations:** ^1^ Mellanby Centre for Musculoskeletal Research, Department of Oncology and Metabolism University of Sheffield Sheffield UK

**Keywords:** ANABOLIC TREATMENT, ANTIRESORPTIVE TREATMENT, DIABETES, OSTEOPOROSIS

## Abstract

Diabetes is characterized by hyperglycemia, but the two main types, type 1 diabetes (T1D) and type 2 diabetes (T2D), have distinct pathophysiology and epidemiological profiles. Individuals with T1D and T2D have an increased risk of fractures, particularly of the hip, upper arm, ankle, and nonvertebral sites. The risk of fractures is higher in T1D compared to T2D. The diagnosis of osteoporosis in individuals with T1D and T2D follows similar criteria as in the general population, but treatment thresholds may differ. Antiresorptive therapies, the first‐line treatment for osteoporosis, are effective in individuals with T2D. Observational studies and post hoc analyses of previous trials have indicated that antiresorptive drugs, such as bisphosphonates and selective estrogen receptor modulators, are equally effective in reducing fracture risk and increasing bone mineral density (BMD) in individuals with and without T2D. Denosumab has shown similar effects on vertebral fracture risk but increases the risk of nonvertebral fractures. Considering the low bone turnover observed in T1D and T2D, anabolic therapies, which promote bone formation and resorption, have emerged as a potential treatment option for bone fragility in this population. Data from observational studies and post hoc analyses of previous trials also showed similar results in increasing BMD and reducing the risk of fractures in people with or without T2D. However, no evidence suggests that anabolic therapy has greater efficacy than antiresorptive drugs. In conclusion, there is an increased risk of fractures in T1D and T2D. Reductions in BMD cannot solely explain the relationship between T1D and T2D and fractures. Bone microarchitecture and other factors play a role. Antiresorptive and anabolic therapies have shown efficacy in reducing fracture risk in individuals with T2D, but the evidence is more robust for antiresorptive drugs. Evidence in T1D is scant. Further research is needed to fully understand the underlying mechanisms and optimize management strategies for bone fragility in T1D and T2D. © 2023 The Authors. *JBMR Plus* published by Wiley Periodicals LLC on behalf of American Society for Bone and Mineral Research.

## Graphical abstract

Type 1 (T1D) and type 2 diabetes (T2D) are associated with an increased risk of fractures. Antiresorptive and anabolic therapies have shown efficacy in reducing fracture risk in individuals with T2D, but evidence is more robust for antiresorptive drugs. Evidence in T1D is scant. Further research is needed to understand the underlying mechanisms and optimize management strategies for bone fragility in T1D and T2D.

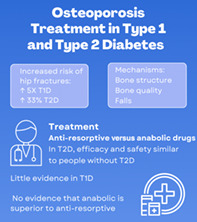

## Introduction

Diabetes mellitus represents a global health burden. Recent studies report that the disease affected 9.3% of the world population (463 million people) in 2019, and estimates suggest it will rise to 10.2% (578 million) by 2030 and 10.9% (700 million) by 2045.^[^ ^1^ ^]^ One in two people living with diabetes does not know they have the disease.^[^ ^1^ ^]^ Hyperglycemia is the hallmark of diabetes, but distinctive pathophysiological and epidemiological profiles characterize the two main types.

Type 1 diabetes (T1D) is characterized by the autoimmune destruction of pancreatic beta cells, resulting in insulin deficiency. T1D primarily affects young individuals. The incidence rates display regional variation, ranging from 1 to 20 cases per 100,000 person‐years, with higher rates observed in developed countries. Conversely, type 2 diabetes (T2D), characterized by insulin resistance and impaired beta‐cell function, typically develops later in life and is strongly associated with lifestyle factors such as obesity and sedentary behavior. Globally, T2D accounts for most diabetes cases, with increasing prevalence across all continents, driven by aging populations, urbanization, and unhealthy lifestyles.^[^ ^1^ ^]^ The epidemiology of the two main types of diabetes underscores the multifaceted interplay between genetic susceptibility, environmental factors, and lifestyle behaviors, emphasizing the need for comprehensive strategies targeting prevention, early detection, and management of these chronic conditions. In this manuscript, we discuss the rationale for the use of antiresorptive, anabolic, and dual‐mechanism antiosteoporosis therapies in T1D and T2D. We review the current literature, examine the evidence, and summarize the key findings on efficacy and safety.

### The risk of fractures in type 1 and type 2 diabetes

T1D and T2D are associated with an increased risk of fractures at most sites investigated. Overall, the risk of hip,^[^ ^2^, ^3^ ^]^ upper arm,^[^ ^3^ ^]^ ankle,^[^ ^3^, ^4^ ^]^ and nonvertebral fractures^[^ ^2^ ^]^ was higher in people with T1D and T2D compared to people without diabetes. The increase in risk is higher in T1D than in T2D.^[^ ^2^, ^3^, ^5^ ^]^ In T1D, the risk of hip fractures is 393% higher,^[^ ^2^ ^]^ while the risk of any fractures was 216% higher and spine fractures 188% higher^[^ ^6^ ^]^ than those without diabetes. Conversely, in T2D, there is a 33% increase in the risk of hip fractures, a 19% increase in the risk of nonvertebral fractures,^[^ ^2^ ^]^ a 54% increase in the risk of upper arm fracture, a 22% increase in any fracture, and a 15% increase in ankle fracture.^[^ ^3^ ^]^ In T2D, the risk was higher in those younger, those using insulin, and those with a longer duration of diabetes.^[^ ^2^ ^]^

### Areal bone mineral density in type 1 and type 2 diabetes

Reductions in bone density do not explain the increased risk of fractures in people with T1D and T2D. The classical pattern reported is that areal bone mineral density (aBMD) is decreased in T1D and normal or increased in T2D.^[^ ^5^ ^]^ Body mass index (BMI) was found to be a major determinant of aBMD, while glycated hemoglobin (HbA1c) was not linked to aBMD.^[^ ^5^ ^]^ More recent studies have found that adults with T1D have a modestly lower aBMD at the femoral neck and lumbar spine than adults without diabetes.^[^ ^7^ ^]^ Due to the early onset of T1D, the hypothesis that the disease could compromise the peak of bone mass accrual was also raised to explain the increased risk of fractures. However, Halper‐Stromberg et al. have compared aBMD across the lifespan in individuals with T1D and age‐ and sex‐matched healthy controls.^[^ ^8^ ^]^ The study found that lumbar spine aBMD was similar in patients with T1D compared with age‐ and sex‐matched participants without diabetes, except in postmenopausal women with T1D who had a lower lumbar spine, femoral neck, and total hip aBMD.^[^ ^8^ ^]^ Therefore, low aBMD cannot fully explain the greater fracture risk observed in adults with T1D.

### Microarchitecture

Several authors have investigated bone microarchitecture in T1D and T2D using high‐resolution peripheral quantitative computed tomography (HR‐pQCT), and the results are conflicting. Recently, a meta‐analysis has summarized the findings and concluded that T1D is associated with adverse trabecular characteristics at the radius, while T2D is associated with favorable trabecular characteristics but adverse cortical features also at the radius.^[^ ^9^ ^]^ Although meta‐analyses are empowered by including several studies and large numbers, it is important to acknowledge that several groups of participants with different characteristics have been included. In the studies that investigated T2D, higher cortical porosity was a common finding, both at the radius^[^ ^10^, ^11^, ^12^, ^13^ ^]^ and tibia,^[^ ^11^, ^12^ ^]^ especially in those participants who had microvascular disease^[^ ^13^ ^]^ or experienced a fracture.^[^ ^14^ ^]^ Higher cortical porosity was also reported at the tibia in participants with T1D and neuropathy.^[^ ^15^ ^]^ Despite these findings in microarchitecture and unfavorable findings in finite element analysis,^[^ ^12^, ^16^ ^]^ the results suggest that compromised microarchitecture contributes to bone fragility in T1D and T2D but does not fully explain the increased risk of fractures observed.

### Other features

Histomorphometry studies comparing samples from T1D and T2D to people without diabetes yield conflicting findings. The results showed no differences in T1D^[^ ^17^ ^]^ and unfavorable findings associated with poor disease control and chronic complications in T2D.^[^ ^18^, ^19^ ^]^ Despite these findings, overall bone resorption and formation markers are low and poorly predictive of fracture risk.^[^ ^20^, ^21^, ^22^, ^23^ ^]^ Therefore, bone turnover is reduced, and there are notable alterations in bone material properties and microstructure. These changes are more pronounced when microvascular complications are present.^[^ ^13^, ^15^, ^16^ ^]^ Developing bone fragility in T1D and T2D involves intricate pathophysiological mechanisms, including hyperglycemia, oxidative stress, and the accumulation of advanced glycation end products (AGEs).^[^ ^24^, ^25^ ^]^ These factors compromise the properties of collagen, promote increased marrow adiposity, release inflammatory factors and adipokines from visceral fat, and potentially affect the function of osteocytes.^[^ ^24^, ^25^ ^]^

A recent study evaluated the gene expression in bone samples from postmenopausal women with or without T2D undergoing hip replacement surgery. The study found that SOST gene expression was upregulated in T2D, but sclerostin levels (the product of the SOST gene) did not differ between people with and without T2D. Sclerostin is a potent inhibitor of the canonical Wnt signaling pathway and therefore a negative regulator of bone formation. They also found increased levels of AGEs in bone samples of people with T2D. They suggested that the accumulation of AGEs may contribute to reduced bone formation and impaired bone quality.^[^ ^26^ ^]^

Other factors, such as treatment‐induced hypoglycemia, certain antidiabetic medications (e.g., thiazolidinediones) directly impacting bone and mineral metabolism, and an elevated risk of falls, contribute to the heightened fracture susceptibility observed in individuals with diabetes mellitus.^[^ ^25^ ^]^

### Osteoporosis diagnosis and management in type 1 and type 2 diabetes

The diagnosis of osteoporosis in adults with T1D and T2D is established based on the presence of fragility fractures and/or low aBMD following the diagnostic criteria.^[^ ^27^ ^]^ However, it is important to differentiate these diagnostic criteria from treatment thresholds. It has been observed that prior fracture history is a strong predictor of future fractures in patients both with and without T1D or T2D.^[^ ^28^ ^]^ Therefore, treatment should be initiated for patients with T1D or T2D who had an osteoporotic fracture. In addition, experts recommend that treatment be considered at more favorable Fracture Risk Assessment Tool (FRAX) and BMD values compared to patients without diabetes^[^ ^27^ ^]^ since both BMD and FRAX may underestimate the fracture risk in individuals with T2D.^[^ ^28^, ^29^ ^]^ In people with T2D, fracture risk at T‐score < −2 is equivalent for nondiabetes at T‐score < −2.5, and a T‐score < −2 should be used to consider treatment. The FRAX prediction tool does not include an option for T2D, and it is suggested that the option for rheumatoid arthritis be ticked as an alternative strategy to account for the increased risk in this population.^[^ ^27^ ^]^

## Treatment Options

Once the need for pharmacological treatment is established, it is important to define which medication to use. This manuscript reviews the rationale for using antiresorptive and anabolic treatments, available evidence, and safety issues.

### Rationale for the use of antiresorptive therapy

Antiresorptive drugs are the most commonly used drug in the treatment of osteoporosis. Bisphosphonates are the first line therapy for postmenopausal osteoporosis.^[^ ^30^ ^]^ Due to the low bone turnover observed in T1D and T2D, there are concerns that antiresorptive therapies, which suppress bone turnover, may not be as effective in preventing bone loss and fracture in patients with T1D and T2D.^[^ ^31^ ^]^ However, this is not supported by the available evidence.

### Evidence for the use of antiresorptive therapy

No trials are explicitly designed to investigate the efficacy of antiosteoporosis drugs in people with T1D or T2D. The current evidence comes from observational studies and post hoc analyses of previous randomized controlled trials (RCTs).

A cohort study from Denmark assessed whether the effect of antiresorptive drugs differed in patients with and without T1D and T2D. The study found that T1D or T2D did not affect the fracture‐preventive potential of bisphosphonates or raloxifene, and the low‐turnover state of T1D and T2D did not hinder the effect of these drugs against osteoporosis.^[^ ^32^ ^]^

The post hoc analyses of several antiosteoporosis trials have suggested that the efficacy and safety of treatment in people with T2D are similar to those without the disease.

The post hoc analyses of trials using alendronate^[^ ^33^ ^]^ and risedronate^[^ ^34^ ^]^ found that these bisphosphonates showed consistent safety and efficacy in suppressing bone turnover and increasing aBMD in osteoporosis patients with and without diabetes. Both drugs reduced the rate of fractures and increased aBMD in participants treated for osteoporosis.

Post hoc analyses of two randomized trials of raloxifene found a reduction in vertebral fractures in women with T2D and women without the disease but no reduction in nonvertebral fractures.^[^ ^35^ ^]^ In the Multiple Outcomes of Raloxifene Evaluation (MORE) study, raloxifene showed greater efficacy in reducing vertebral fractures in these patients compared with those without diabetes.^[^ ^36^ ^]^

The effect of denosumab in postmenopausal women with osteoporosis and T2D was assessed in a post hoc analysis of the subgroup with T2D of the Fracture REduction Evaluation of Denosumab in Osteoporosis every 6 Months (FREEDOM) study and its long‐term Extension. Denosumab significantly increased BMD and decreased vertebral fracture risk in women with osteoporosis and T2D; however, nonvertebral fracture incidence was higher with denosumab than placebo in subjects with T2D (11.7% versus 5.9%, *p* = 0.046).^[^ ^37^ ^]^ Fractures at the ribs (*n* = 8) and ulna (*n* = 4) were observed only in those participants with T2D taking denosumab. The numbers of fractures at the radius and humerus were greater in the participants with T2D (8 versus 2 and 7 versus 5, respectively), although there were fewer hip fractures with denosumab than placebo (1 versus 4). However, during the first 3 years of FREEDOM Extension, new vertebral and nonvertebral fracture incidences were low in the long‐term and crossover denosumab groups with T2D (≤6%), consistent with the overall extension population; yearly nonvertebral fracture incidence was comparable to that of the FREEDOM placebo group.^[^ ^37^ ^]^

**Fig. 1:**
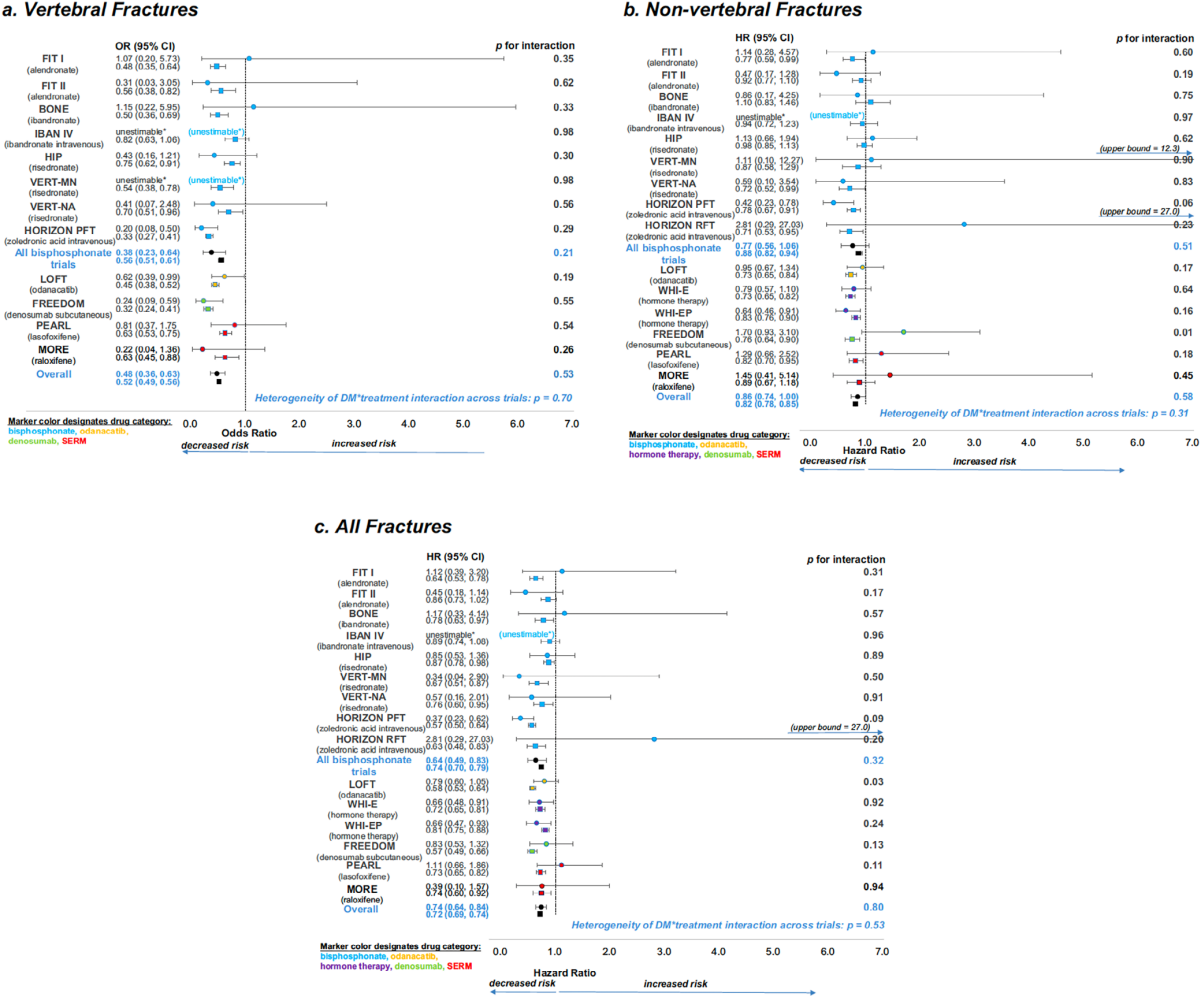
Forest plots showing effects of treatment on fracture risk in T2D (solid circle) and non‐type 2 diabetes (solid square). (*A*) Vertebral fractures, (*B*) nonvertebral fractures, and (*C*) all fractures. The *p* values for T2D status–treatment interaction for each trial, the overall effects, and the *p* value for heterogeneity of T2D status–treatment interaction across trials are all shown. Reproduced with permission from Eastell et al.^[^ ^38^ ^]^

The most robust evidence of the efficacy of antiresorptive therapies comes from the pooled analysis of individual participant data using the Foundation for the National Institutes of Health (FNIH)‐American Society for Bone and Mineral Research (ASBMR)‐Study to Advance Bone Mineral Density (BMD) as a Regulatory Endpoint (SABRE) cohort.^[^ ^38^ ^]^ This unique dataset of individual patient data from randomized, placebo‐controlled trials of osteoporosis therapies included data from 96,385 subjects, 6.8% of whom had T2D, from nine bisphosphonate trials, two selective estrogen receptor modulator (SERM) trials, two trials of menopausal hormone therapy, one denosumab trial, and one odanacatib trial. The group used Cox regression to obtain the treatment‐related hazard ratio (HR) for incident nonvertebral, hip, and all fractures and logistic regression to obtain the treatment‐related odds ratio (OR) for incident radiographic vertebral fractures, separately for T2D and people without diabetes. Linear regression was used to estimate the effect of treatment on the 2‐year change in BMD (*n* = 49,099) by diabetes status (T2D). In all analyses, the interaction between treatment and diabetes status was assessed. In pooled analyses of all 15 trials, it was found that T2D did not impact treatment efficacy, with similar reductions in vertebral, nonvertebral, all, and hip fractures and similar increases in lumbar spine, total hip, and femoral neck aBMD for the drugs licensed to treat osteoporosis (Fig. 1). They found similar results for the pooled analysis, including only the bisphosphonate trials. However, when they considered trials individually, they found an interaction between T2D status and the effects of denosumab on nonvertebral fracture risk, consistent with the data reported in the FREEDOM trial post hoc analysis. Despite the low baseline bone turnover observed in patients with T2D, this analysis showed that antiresorptive treatment led to a similar reduction in bone turnover markers in people with and without T2D.^[^ ^38^ ^]^ Thus, the study provides evidence that bisphosphonates and most licensed antiresorptive drugs effectively reduce fracture risk and increase aBMD, irrespective of T2D status. However, the study also highlights the need for further research into the effects of antiresorptive treatments on patients with T2D, especially for individual therapies. Therefore, clinical trials of antiosteoporosis drugs in people with T2D are needed.

### Safety of antiresorptive therapy

Bisphosphonates are generally well tolerated, exhibiting predominantly nonsevere adverse effects. Nevertheless, gastrointestinal complications, namely reflux and esophageal inflammation, are commonly observed. Notably, gastric discomfort represents a frequently encountered side effect associated with bisphosphonates, and in more severe instances, it may give rise to erosions on the esophageal epithelium. To avoid the occurrence of these adverse effects, it is recommended to maintain an upright position for 30–60 min after the oral administration of bisphosphonates.^[^ ^30^ ^]^

Emerging evidence suggests an association between long‐term antiresorptive use, especially bisphosphonates, and an increased risk of atypical femur fractures (AFFs). These fractures are characterized by transverse or oblique patterns and minimal or no trauma history and occur at the subtrochanteric and diaphyseal regions of the femur. The underlying mechanisms responsible for this association remain unclear, although hypotheses have been proposed, including the suppression of bone remodeling and the accumulation of microdamage.^[^ ^39^ ^]^ Since these features are also observed in T1D and T2D, there is concern about the risk of these fractures in these populations.

Data from the Danish National Patient Register showed that patients with T1D had a higher risk of subtrochanteric and femoral shaft fractures compared to the general population. The 19,896 patients with T1D, 312,188 patients with T2D, and 996,252 controls were followed from 1996 to 2017. There was no increased risk in patients with T2D. Previous fractures and the use of bisphosphonates were associated with an increased risk of these fractures. However, the study was not able to characterize the subtrochanteric and femoral shaft fractures as AFFs.^[^ ^40^ ^]^

In another study where AFFs were characterized, approximately half of AFFs were not associated with bisphosphonate use. There were no significant demographic or clinical differences between bisphosphonate and nonbisphosphonate‐related AFFs, including T2D.^[^ ^41^ ^]^ Finally, in another cohort, the risk of AFFs increased with a longer duration of bisphosphonate use, Asian ancestry, shorter stature, overweight, and glucocorticoid use. Hip BMD, T1D, and T2D were not found to be associated with AFF risk.^[^ ^42^ ^]^ Therefore, despite potential common features, there is no strong evidence that the use of bisphosphonates in T1D or T2D is associated with AFF.

T1D and T2D are considered risk factors for medication‐related osteonecrosis of the jaw (MRONJ), a condition characterized by nonhealing exposed bone in the jaw associated with antiresorptive therapy.^[^ ^43^ ^]^ There is no consensus on the role of T1D or T2D in the development of MRONJ. Microvascular ischemia, endothelial cell dysfunction, reduced bone remodeling, and increased apoptosis of bone cells related to T1D and T2D could contribute to the development of MRONJ.^[^ ^44^ ^]^ While MRONJ has been associated with hyperglycemia,^[^ ^45^, ^46^ ^]^ studies have produced conflicting results regarding the prevalence of T1D and T2D among individuals with MRONJ. Some observational studies have not reported a higher prevalence of T2D,^[^ ^45^, ^46^ ^]^ while others have shown an increased prevalence.^[^ ^44^, ^47^ ^]^ Therefore, further research is warranted to elucidate the underlying mechanisms and clarify the association between hyperglycemia and the development of MRONJ in individuals with T1D and T2D. Both bisphosphonates and denosumab are associated with an increased incidence of MRONJ. Despite an increase in the relative risk, the absolute incidence of MRONJ is low in patients using antiresorptive drugs to treat osteoporosis, and most cases of MRONJ are associated with high doses of antiresorptive drugs used in the treatment of cancer.^[^ ^43^ ^]^

### Rationale for the use of anabolic therapy

Considering the disproportionately high increase in the risk of fractures compared to the observed effects on bone structure and the low bone turnover observed in people with T1D and T2D, anabolic therapies emerge as an attractive choice. Bone fragility in T1D and T2D is characterized by a low bone turnover state, in contrast to postmenopausal osteoporosis, which is characterized by increased bone turnover. In this scenario, anabolic therapies would increase bone formation and resorption, allowing bone renewal, improving bone material properties, and repairing microcracks. Therefore, we review the current evidence on using anabolic therapies for treating bone fragility in T1D and T2D.

### Evidence for the use of anabolic therapy

In a T2D animal model, T2D significantly decreased aBMD, impaired bone formation, compromised microarchitecture, increased cortical porosity, and reduced bone strength. In this model, treatment with teriparatide and abaloparatide effectively restored aBMD and corrected the deteriorated bone architecture.^[^ ^48^ ^]^ Mechanistically, teriparatide and abaloparatide induced similar responses at the tissue and gene signature levels, promoting bone formation and resorption with a positive balance favoring bone gain. Both agents demonstrated the ability to restore bone architecture, correct cortical porosity, and improve the mechanical properties of bone in animals with T2D. Notably, abaloparatide treatment resulted in increased toughness, indicating enhanced fracture resistance. Furthermore, both agents increased bone strength, even in the presence of severe hyperglycemia, surpassing the strength observed in healthy controls.^[^ ^48^ ^]^

There is no specific trial on the efficacy and safety of anabolic treatments for osteoporosis in humans with T1D or T2D. The evidence in T2D is limited to observational studies and post hoc analyses of osteoporosis RCTs.

A post hoc analysis of the Abaloparatide Comparator Trial In Vertebral Endpoints (ACTIVE) evaluated the efficacy and safety of abaloparatide in women with T2D. This phase 3 trial included 198 participants with T2D and compared abaloparatide, teriparatide, and placebo. Abaloparatide and teriparatide treatments demonstrated significant improvements in aBMD and trabecular bone score (TBS) compared to placebo, consistent with the overall trial population. The subgroup analysis of the RCT was not powered to assess fractures, but fracture events were reduced with abaloparatide treatment in T2D patients, especially for nonvertebral fractures, where there was a significant reduction in fractures when compared to placebo. Safety outcomes were similar to those in the overall trial population. These findings suggest that abaloparatide may effectively reduce fracture risk in patients with T2D.^[^ ^49^ ^]^

**Fig. 2:**
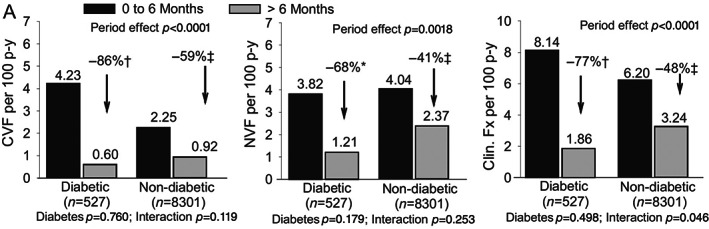
Fracture rates by diabetes mellitus presence or prior bisphosphonate use at baseline and treatment period. Shown are clinical vertebral fracture (left), nonvertebral (middle), and clinical fracture (right) rates per 100 patient‐years for the reference period (0–6 months) versus the postreference period (>6 months) for subgroups based on diabetes mellitus presence at baseline. *p < 0.05; †*p* < 0.005; ‡*p* < 0.0001 between periods. Time effect compares fracture rate between the two treatment periods irrespective of subgroup; interaction assesses whether time effect varied between subgroups; subgroup compares fracture rate between subgroups irrespective of period effect. Period and subgroup significant at *p* < 0.05; interaction significant at *p* < 0.10. CVF, clinical vertebral fracture; Fx, fractures; Gluc., glucocorticoid; NVF, nonvertebral fracture; pBisph., prior bisphosphonate use at baseline; p‐y, patient‐years of treatment; RA, rheumatoid arthritis. Reproduced with permission from Langdahl et al.^[^ ^51^ ^]^

Another post hoc analysis has explored the effects of teriparatide on T2D using real‐world data: the Direct Analysis of Nonvertebral Fractures in the Community Experience (DANCE) study. The study found a similar reduction in nonvertebral fracture incidence, increased aBMD, and decreased back pain in patients with and without T2D.^[^ ^50^ ^]^ Another study, which included data from four observational studies (including the DANCE study), found significant reductions in clinical vertebral fractures, nonvertebral fractures, clinical fractures, and hip fractures during teriparatide treatment in people with T2D compared to people who did not receive treatment. The study suggests that for clinical fractures, participants with T2D responded better to teriparatide than those without diabetes (Fig. 2).^[^ ^51^ ^]^ Therefore, current evidence suggests that anabolic therapies have at least similar efficacy in patients with T2D compared to people without the disease. However, this evidence comes from observational studies and post hoc analyses; specific trials addressing this population are needed.

### Safety of anabolic therapy

There are no specific concerns regarding the use of anabolic treatments in people with diabetes.

## Evidence in T1D

Evidence in T1D is scant.^[^ ^52^ ^]^ In a mouse model, T1D was associated with bone loss and decreased osteoblast activity and viability. Bisphosphonate therapy, commonly used to treat osteoporosis, inhibits osteoclast activity and osteoblast apoptosis. The study on mice found that weekly alendronate treatment prevented T1D‐induced osteoblast death and trabecular bone loss. Alendronate also reduced marrow adiposity and increased bone stiffness but decreased the work required for fracture in mice with T1D. In addition, longer treatment suppressed bone formation and osteoblast markers in mice with T1D.^[^ ^53^ ^]^ Patients with T1D are usually excluded from RCTs. The Danish observational cohort included data on T1D and T2D and concluded that the disease does not affect the fracture prevention potential of bisphosphonates or raloxifene. The analysis was not stratified by diabetes type, but when the authors compared the effect of osteoporosis drugs on the risk of hip fractures, there was no difference between T1D and T2D and people without diabetes.^[^ ^32^ ^]^ Another observational study has reported the effect of risedronate (30 mg/week) in patients with T1D with osteoporosis or osteopenia. Risedronate use associated with calcium and vitamin D (*n* = 35) resulted in an increase in lumbar spine and femoral neck aBMD after 12 months in patients with osteoporosis, while no difference was observed in patients who received only calcium and vitamin D (*n* = 17).^[^ ^54^ ^]^ In light of the lack of evidence regarding osteoporosis treatment in patients with T1D, current practice is based on osteoporosis guidelines and data available from T2D. Data on T1D are urgently needed to guide treatment in this population at high risk of fractures.

## Dual Mechanism of Action

### Rationale for the use of romosozumab

Sclerostin is involved in the bone's adaptive response to mechanical loading. Osteocytes produce sclerostin, which has a dual action: it inhibits the canonical Wnt pathway, resulting in the suppression of osteoblast activity, and simultaneously stimulates the release of receptor activator of NF‐κB ligand by osteocytes, thereby promoting osteoclast recruitment. Consequently, the inhibition of sclerostin leads to the stimulation of bone formation and the inhibition of bone resorption. Clinical trials investigating romosozumab, an antibody targeting sclerostin, have shown its efficacy in enhancing BMD and reducing fracture risk when compared to both placebo and alendronate.^[^ ^55^, ^56^ ^]^

In animal models, sclerostin antibody treatment reverses the adverse effects of T2D on bone mass and strength and improves bone defect regeneration.^[^ ^48^, ^57^ ^]^ Previous meta‐analysis reported increased sclerostin in both T1D and T2D.^[^ ^22^ ^]^ In addition, sclerostin gene expression was reported to be higher in bone samples of people with T2D than those without the disease.^[^ ^26^ ^]^ These data suggest that sclerostin might be involved in bone fragility in T1D and T2D, making antisclerostin antibodies a candidate to treat bone fragility in the disease.

### Evidence for the use of romosozumab

There is no evidence regarding the efficacy and safety of romosozumab in patients with T1D or T2D.

### Safety of romosozumab in T1D and T2D

Concerns have been raised regarding the cardiovascular safety of romosozumab, and its use is not recommended in patients with high cardiovascular risk.^[^ ^58^ ^]^ Since T1D and T2D are associated with an increased risk of cardiovascular events, it is unlikely that the benefits of using romosozumab would outweigh the risk of increasing the cardiovascular risk in this population.

## Conclusion

Available evidence suggests that antiresorptive and anabolic therapies have similar effects on bone density and fracture risk reduction in patients with and without T2D. However, the evidence for T2D is limited to observational studies and post hoc analyses of osteoporosis RCTs, and there is scant evidence for T1D. There is no clinical evidence for the use of antisclerostin antibodies in T1D and T2D. Prospective studies evaluating the effect of available therapies on bone quality and fracture outcomes in patients with T1D and T2D are needed. Despite the rationale that favors the use of anabolic agents to treat people with T1D and T2D due to the low bone turnover observed, this is not supported by current evidence. Studies comparing the effects of bisphosphonates and anabolic agents on people with T1D and T2D would be required to support this practice.

## Author Contributions

**Tatiane Vilaca:** Conceptualization; methodology; writing – original draft; writing – review and editing. **Richard Eastell:** Conceptualization; writing – review and editing.

## Funding Information

The authors received no funding for this project.

## Disclosures

Richard Eastell receives consultancy funding from Immunodiagnostic Systems, Sandoz, Samsung, CL Bio, Biocon, Takeda, UCB, meeting presentations for Pharmacosmos, Alexion, UCB, and Amgen, and grant funding from Alexion. Tatiane Vilaca received consultancy and grant funding from Pharmacosmos and grant funding from Alexion.

### Peer Review

The peer review history for this article is available at https://www.webofscience.com/api/gateway/wos/peer-review/10.1002/jbm4.10838.

## Data Availability

No datasets were generated or analyzed during the current study. The review is based on the references cited.
